# Role of BMEC‐Derived Exosomal miR‐144‐3p in Microglial Polarization: Unveiling the NLRP3‐GSDMD Pathway in Intracerebral Hemorrhage

**DOI:** 10.1155/mi/6142465

**Published:** 2026-05-26

**Authors:** Jun Ma, Zhongying Ran, Tao Luo, Zexin Jin, Ying Tan, Hao Yin

**Affiliations:** ^1^ Department of Neurosurgery, Guizhou Provincial People’s Hospital, Guiyang, China, 5055.cn

**Keywords:** inflammation, intracerebral hemorrhage, M1/M2 polarization, miR-144-3p

## Abstract

**Background:**

Intracerebral hemorrhage (ICH) often triggers a cascade of inflammatory responses, leading to secondary brain injury. The present work elucidated the regulatory mechanisms of NLRP3 inflammasome pathway activation in exosomal vesicle secretion by brain microvascular endothelial cells (BMECs)‐derived exosomes and its influence on the polarization and recruitment of microglial cells (GCs).

**Methods:**

In an in vitro model, BMECs were pretreated with hemin to mimic the ICH environment and then cocultured with GCs in a replaced medium. NLRP3 inhibitor MCC950 and exosome release inhibitor GW4869 were used to observe the effects of NLRP3 inflammation and exosomes in BMECs. Gene expression was detected using western blotting (WB), immunofluorescence (IF), and real‐time quantitative PCR. M1 polarization and migration of GCs were evaluated using biomarker expression, flow cytometry, and Transwell assays. The cellular mechanisms were further explored using an in vivo ICH rat model.

**Results:**

Hemin pretreatment activated NLRP3 inflammasomes and upregulated gasdermin D (GSDMD‐N) production in BMECs, which further contributed to enhanced migration and M1 polarization of cocultured GCs. MCC950 and GW4869 significantly inhibited the effects of BMECs on GCs migration and M1 polarization. Mechanically, the NLRP3/GSDMD pathway promoted the release of exosomes from BMECs, which were absorbed by GCs and resulted in changes in their migration ability and polarization. Furthermore, miR‐144‐3p was identified as an important regulator in BMEC‐derived exosomes and mediated the effect of BMECs on GSDMD. Knockdown of GSDMD in BMECs weakened the enhanced migration and M1 polarization of GCs by inhibiting the release of BMEC‐derived exosomes, while miR‐144‐3p overexpression in GCs abolished these effects. An in vivo study illustrated that MCC950 decreased pro‐inflammatory cytokine and CD86 levels, which were recovered by miR‐144‐3p overexpressing adeno‐associated virus (AAV) injection.

**Conclusions:**

This investigation revealed that the NLRP3/GSDMD pathway promotes the secretion of BMEC‐derived exosomes, which transfer miR‐144‐3p from BMECs to GCs, further inducing the migration and M1 polarization of GCs.

**Summary**

• NLRP3/gasdermin D (GSDMD) promotes the release of exosomes from brain microvascular endothelial cells (BMECs).
• BMECs transfer miR‐144‐3p to GCs through exosomes.
• miR‐144‐3p exacerbates the M1 polarization and migration of GCs.

## 1. Introduction

Intracerebral hemorrhage (ICH) represents one of the most catastrophic forms of stroke, characterized by exceptionally poor clinical outcomes, inducing a 5‐year mortality rate of 70% and severe disability affecting more than 80% of survivors [1]. Unfortunately, its incidence has increased with the growing elderly population [2]. However, no effective therapeutic methods have been employed in clinics to date. Despite extensive research efforts in recent years to uncover the molecular basis of ICH and discover novel therapeutic targets, effective treatment strategies remain limited.

As the primary structural and functional constituents of the blood–brain barrier (BBB), brain microvascular endothelial cells (BMECs) play a critical role in maintaining cerebral homeostasis. Their dysfunction has been implicated in various neurological pathologies, including cerebral edema, ICH, and brain tumor progression. Primary cultured BMECs have been widely employed for studying cerebrovascular diseases, including ICH, because of their high similarity to in vivo cells. Microglia, the resident macrophage population of the central nervous system (CNS), serve as the primary immune defense mechanism to clear damaged nerves, plaques, and infectious substances in the CNS. When damage and pathological stress occur, microglial cells polarize into M1 or M2 phenotypes. Microglia in the M1 phenotype release pro‐inflammatory cytokines and neurotoxic molecules that promote inflammation and cytotoxic reactions [3, 4], whereas the M2 phenotype acts oppositely. Therefore, M1/M2 polarization is critical for regulating inflammation. In ICH, inflammation is considered one of the most critical factors inducing a poor overall prognosis [5]. Numerous studies have focused on the molecular mechanisms of M1/M2 polarization in ICH. For example, some noncoding RNA molecules, such as microRNA (miRNA)‐494 [6], miRNA‐575 [7], and NEAT1 [8], have been reported to regulate M1/M2 polarization in ICH. Consequently, precise modulation of the transition between microglial M1 (proinflammatory) and M2 (anti‐inflammatory) activation states represents a significant therapeutic challenge in managing neuroinflammatory disorders.

As a subset of extracellular vesicles (EV) ranging from 30–150 nm in diameter, exosomes are secreted by virtually all cell types under both normal and pathological conditions. These nano‐sized vesicles mediate crucial biological processes through their cargo of regulatory molecules, including microRNAs, and play essential roles in cell communication and epigenetic regulation [9, 10]. Emerging evidence reveals the significant involvement of the miRNAs derived from BMEC‐secreted exosomes in the pathophysiology of stroke and ischemia [11]. Zhang et al. [12] identified miR‐36133p from ischemic BMECs as a potent inducer of microglial M1 polarization. This finding aligns with the established regulatory role of exosomal miRNAs in modulating microglial phenotypic switching between M1/M2 states [13, 14]. However, studies on how exosomal miRNAs regulate microglial M1 polarization by BMECs in ICH remain unclear.

The NLRP3 inflammasome represents a multiprotein complex comprising three core components: the NLRP3 sensor molecule, the adaptor protein ASC (apoptosis‐associated speck‐like protein containing a caspase recruitment domain), and the effector protease caspase‐1. The NLRP3 inflammasome undergoes activation through diverse stimuli, including pathogen‐associated molecular patterns (PAMPs) and damage‐associated molecular patterns (DAMPs), culminating in the maturation and secretion of pro‐inflammatory cytokines interleukin (IL)‐1β and IL‐18. This process is mechanistically linked to gasdermin D (GSDMD), a pore‐forming protein that executes pyroptotic cell death – a highly inflammatory form of programmed cell death characteristic of innate immune responses. Upon activation, caspase‐1 executes dual substrate cleavage: converting cytokine precursors to mature IL‐1β/IL‐18 while simultaneously processing full‐length GSDMD into its functional N‐terminal and regulatory C‐terminal fragments, thereby coupling inflammatory signaling with pyroptotic cell death. GSDMD‐N can be inserted into the cell membrane to form pores, thereby inducing pyroptosis in cells. Previous studies have indicated that NLRP3/GSMD is correlated with enhanced EVs release, and these EVs exaggerate inflammation, the mechanism of which is unclear [15]. Recently, it has been revealed that GSDMD promotes the loading of inflammatory factors into EVs, and the pores in the cell membrane induced by GSDMD serve as a channel to accelerate EV release [16, 17]. Mouasni et al. [18] found that the activation of the classical NLRP3 inflammasome significantly promotes the secretion of Fas‐associated death domain (FADD) in a vesicular manner, participating in cell death, proliferation, innate immunity, and inflammation, as seen in rheumatoid arthritis.

miRNAs represent a class of endogenous noncoding RNA molecules, typically 21–22 nucleotides in length. They exert their function in gene regulation predominantly by directly targeting mRNAs, leading to either mRNA cleavage or translational repression [19]. miR‐144 is a strand of miRNA located on the 17th chromosome in humans, exhibiting high conservation in mice and rats. miR‐144‐3p has been identified as a critical regulatory molecule influencing the pathogenesis of multiple inflammatory disorders. Current evidence demonstrates its involvement in modulating key inflammatory pathways across various disease states. Clinical investigations have demonstrated upregulated miR‐144‐3p in peripheral blood monocytes during bacterial infection, showing significant positive correlations with pro‐inflammatory cytokine levels (IL‐1β and IL‐6). The observed strong correlation between miR‐144 expression and macrophage infiltration in pulmonary tissues implies that this miRNA may participate in the chemotactic signaling pathways governing monocyte migration and tissue retention [20]. Laboratory data suggest that heme treatment markedly upregulates the miR‐144‐3p level in leukocytes, triggering an overactive inflammatory response [21]. Notably, circulating exosomal miR‐144‐3p levels were significantly elevated in serum samples from Crohn’s disease patients [22]. In contrast, miR‐144‐3p modulates macrophage polarization, thereby participating in immune regulation. Following bacterial infection, macrophages infiltrate the lungs and adjust the local inflammatory response [20].

This study systematically investigates the regulatory role of miR‐144‐3p in modulating microglial polarization dynamics (M1/M2) and neuroinflammation responses following ICH, aiming to delineate the molecular pathways underlying posthemorrhagic inflammation.

## 2. Materials and Methods

### 2.1. ICH Animal Model Establishment

Forty adult male Sprague‐Dawley rats (weighing 300–400 g and 3–4 months old) were provided by the Animal Experimental Center of Guizhou Medical University. Animals were acclimatized for ≥7 days in standard pathogen‐free conditions under a 12:12 h light–dark cycle with free access to water and food. The rats were blindly assigned to five groups (eight rats per group) based on the experimental design using the random number table method. The study was approved by the ethics committee of Guizhou Provincial People’s Hospital (Number SYXK2018‐0003).

The in vivo ICH model was constructed using the ICH method described by Kung et al. [23] with minor differences. The ICH model was established via stereotaxic intrastriatal injection of collagenase type IV. Preoperatively, rats underwent 12 h of fasting with 4 h of water restriction to stabilize metabolic parameters. Surgical anesthesia was induced with intraperitoneal 0.4% sodium pentobarbital and maintained under aseptic conditions. Following anesthesia induction, a T‐shaped scalp incision was made to expose the skull over the right striatum. Using a stereotaxic apparatus, a 3 mm × 1 mm × 5 mm burr hole was drilled. Then, a Hamilton microsyringe was then used to slowly infuse collagenase IV solution into the striatum over 5 min. The needle was maintained in position for 2 min postinjection to prevent reflux before being gradually withdrawn. The hand was gradually withdrawn, and the skull was sealed. Finally, the burr hole was sealed, and the skin was sutured to protect the incision. Postoperatively, the rats were monitored in temperature‐controlled ventilated cages until full recovery of ambulation. The ICH model group received a stereotaxic intrastriatal injection of 2.0 μL collagenase/heparin, while the sham control group received an equivalent volume of sterile normal saline.

To observe the function of miR‐144‐3p and NRLP3 in vivo, an adeno‐associated virus (AAV) and the NLRP3 inhibitor MCC950 were employed. AAV overexpressing miR‐144‐3p (miR‐144‐3p sequence: UACAGUAUAGAUGAUGUACU) as well as empty vector were procured from Hanbio Biotechnology (Hanbio, China). Three weeks before ICH model construction, 5 μL of AVV was injected into the striatum of rats [24]. Half of 1 h after ICH model establishment, 1 μL of MCC950 (0.2 mM) or normal saline was applied to the same site [25].

### 2.2. Functional Assessment

Neurological deficits were quantitatively assessed using the modified neurological severity score (mNSS) [26]. Scores ranged from 0 (normal) to 18. The severity of neurological function damage in different groups was divided into three levels according to the scores: mild dysfunction (scoring 1–6 points), moderate impairment (7–12 points), and severe neurological deficits (13–18 points).

### 2.3. Hemorrhagic Volume Detection

A spectrophotometric method was used to evaluate the hemorrhagic volume [27]. Briefly, the brain tissue was mechanically homogenized in lysis buffer and subjected to high‐speed centrifugation (15,000 × *g*, 30 min, 4°C) to obtain clarified supernatants. The hemoglobin content was determined spectrophotometrically through reaction with Drabkin’s reagent (Sigma–Aldrich), after which the optical density (OD 540 nm) was measured using spectrophotometry (OptiMax; Molecular Devices, Sunnyvale, CA).

### 2.4. Hematoxylin–Eosin (HE) and Immunohistochemical (IHC) Staining

The tissues were precooled in heparinized physiological saline and 4% paraformaldehyde. Brain samples were collected, paraffin‐embedded, and sectioned at 4 μm coronally. Tissue samples from the lesion core were processed for HE staining (Solarbio, Beijing, China) following standard protocols.

For IHC staining, brain tissue samples underwent standard preparation involving fixation in 4% paraformaldehyde, paraffin embedding, and sectioning. After deparaffinization with xylene and graded alcohol rehydration, sections were subjected to antigen retrieval before overnight incubation at 4°C with specific primary antibodies against F4/80 (ab213200, Abcam, Cambridge, MA, USA), CD68 (ab283654), and CD163 (ab182422). The next day, tissue sections were probed with HRP‐labeled goat anti‐rabbit IgG secondary antibody (ab302644). Immunopositive cells were subsequently calculated using light microscopy (Nikon Eclipse 80i).

### 2.5. Primary Microglial Culture and Transfections

Primary microglia were isolated from neonatal rats [28]. Briefly, cortical tissues from newborn rats were dissected, minced, and digested. The mixed cells were then plated in a flask and maintained in complete medium (DMEM with 20% FBS and 1% P/S), with regular medium changes every third day. Microglial cells were subsequently isolated through gentle mechanical agitation from the mixed glial cultures after 10 days of incubation. Following centrifugation at 1000 rpm for 5 min, the harvested microglia were resuspended in DMEM/F12 medium supplemented with 10% FBS and 1% P/S and cultured under standard conditions (37°C, 5% CO_2_). The isolated microglia were identified by immunofluorescence (IF) staining of the biomarker IBA1, and the purity of the microglia was over 90%.

The cerebral cortices of postnatal day 0–1 SD rats were aseptically collected and processed for BMEC isolation. Tissues were initially washed three times with ice‐cold D‐Hanks balanced salt solution, followed by mechanical dissociation into 1–2 mm^3^ fragments in serum‐free DMEM. The tissue fragments were enzymatically digested using a 0.1% collagenase II solution (supplemented with 30 U/mL DNase I; 1 mL per brain equivalent) at 37°C for 90 min with gentle agitation. The digested suspension was subsequently mixed with 20% BSA and subjected to density gradient centrifugation (1000 × *g*, 20 min, 4°C). The vascular‐rich pellet was collected and further digested with a 0.1% collagenase/dispase blend (containing 20 U/mL DNase I) for 60 min at 37°C. After repeat centrifugation (1000 × *g*, 20 min), the final cellular precipitate was washed with DMEM and cultured with 20% FBS and 100 μg/mL heparin sodium at 37°C and 5% CO_2_. The obtained BMECs were identified by IF staining of the biomarker von Willebrand factor, and the purity of BMECs was over 90%.

An in vitro ICH model was constructed using rat BMECs treated with 0, 10, 20, and 40 μM hemin for 24 h. The culture medium was removed, and the treated cells were cocultured with microglia. Moreover, BMECs were pretreated with the NLRP3 inhibitor MCC950 (20 μM) or GW4869 (10 μM) for 24 h. To explore the function of the gene, gene overexpression and knockdown were constructed by overexpressing plasmids and siRNA. Negative control (NC), siRNA targeting NLRP3 (targeting sequence: GCCGTATCTGGTTGTGTTA), siRNA targeting GSDMD (targeting sequence: CCACAACATCTTCATATCC), NLRP3 overexpressing plasmid (NM_001191642.1), and miR‐144‐3p mimics (miR‐144‐3p sequence: UACAGUAUAGAUGAUGUACU) and corresponding NC were provided by Suzhou Genepharma (China). For cellular transfection, Lipofectamine 2000 transfection reagent (Thermo Fisher Scientific) was used according to the manufacturer’s protocol. Two days later, the cells were collected for further analysis.

### 2.6. Cytotoxicity Detection

The cytotoxicity of hemin was assessed using a CCK‐8 assay. Cells were seeded into 96‐well plates, treated with various concentrations of hemin for 24 h, and then incubated with CCK‐8 solution for 2 h after replacing the culture medium. Absorbance was measured at 450 nm using a microplate reader (Thermo Fisher, Waltham, MA, USA), with the values representing cell viability.

### 2.7. Exosomes Isolation

Endothelial cells from ICH models were processed for exosome collection. Cellular components were initially cleared by triple washing with serum‐depleted medium, followed by sequential centrifugation steps to eliminate cellular fragments and microvesicles. Exosome enrichment was performed using a commercial exosome precipitation reagent (Invitrogen, USA) following the supplier’s recommended procedures. The purified exosomal fractions underwent phosphate‐buffered saline (PBS) washing and sterile filtration through 0.22 μm PVDF membranes (Millipore) to ensure particle homogeneity.

### 2.8. Ultrastructural Analysis of Exosomes by Transmission Electron Microscopy (TEM)

Purified exosome samples were resuspended in PBS and incubated overnight at 4°C for stabilization. Subsequently, the exosome solution was loaded onto 200‐mesh copper grids, allowed to adhere for 20 min, and washed thrice. Negative staining was performed using 2% uranyl acetate solution (30 s incubation), followed by air‐drying. Samples were visualized under a Hitachi HT‐7800 transmission electron microscope (Hitachi High‐Technologies, Japan) operating at an acceleration voltage of 100 kV.

### 2.9. Nanoparticle Tracking Analysis (NTA)

NTA was performed to characterize exosome size distribution and concentration. Purified exosomes were diluted in PBS to achieve an optimal particle density of 20–100 per field of view. Triplicate measurements were conducted at 25°C using a Nanosight NS300 system (Malvern Panalytical) configured with camera level 13, 30 s acquisition time, and detection threshold set to 7. Each measurement included the analysis of ≥200 completed particle tracks. Data acquisition and analysis were performed using NTA software (Version 3.2, Malvern Instruments), which calculated particle parameters based on Brownian motion dynamics.

### 2.10. Western Blotting (WB)

Cell and tissue proteins were lysed using RIPA lysis buffer (#R0278, Sigma) and quantified using a BCA Protein Assay Kit (Beyotime, Jiangsu, China). Samples were loaded onto Tris‐glycine SDS sample buffer (Novex) under 100 V and a concentration gel under 120 V. Following electrophoresis, protein bands were electrophoretically transferred to polyvinylidene fluoride membranes using standard protocols. The membranes were then incubated in blocking solution (5% nonfat milk in TBS‐Tween 20) with constant shaking to minimize nonspecific binding. Primary antibodies of NLRP3 (1:2000, ab263899), PYCARD (1:1000, ab309497), CD9 (1:1000, ab307085), TSG101 (1:2000, ab125011), iNOS (1:1000, ab178945), Arg‐1 (1:1000, ab315110), and GAPDH (1:5000, ab8245) obtained from Abcam were employed. Following blocking, membranes were probed with primary antibodies for 60 min at room temperature, followed by washing with TBST. Subsequently, membranes were incubated with HRP‐conjugated goat anti‐rabbit IgG secondary antibody (S0001, Affinity Biosciences, Cincinnati, OH, United States). After the experiment, immunoreactive bands were visualized using an enhanced chemiluminescence (ECL) substrate (Beyotime, Shanghai, China) with a 60 s exposure time. Protein signals were captured using a chemiluminescence imaging system (36209ES01, Qcbio Science & Technologies, Shanghai) and quantified using Image‐Pro Plus 6.0 software. Band intensities were normalized to the corresponding GAPDH levels as an internal control. All experimental conditions were independently replicated three times to ensure reproducibility.

### 2.11. IF Analysis

After the cells reached 60%–70%, they were fixed in paraformaldehyde and achieved with Triton X‐100 (1%), and then cultured with primary anti‐NLRP3 and secondary anti‐FITC antibodies (Sigma, Darmstadt, DE). The cells were then stained with 4^′^, 6‐diamidino‐2‐phenylindole (DAPI) and examined under a confocal laser microscope (Olympus Optical, Tokyo, Japan).

### 2.12. Enzyme‐Linked Immunosorbent Assay (ELISA) Analysis

Tissue and plasma levels of proinflammatory factors were measured in tissues and plasma using commercially available ELISA kits according to the standardized protocol. First, samples were incubated with HRP‐labeled antibody at 37°C for 60 min. Subsequently, 100 µL of the TMB substrate was added to the culture and incubated for 30 min at 25°C in the dark. Finally, after the Stop Solution was added, the absorbance at 405 nm was measured using an ELISA instrument (Thermo Fisher Scientific Inc.).

### 2.13. Transwell Assay

Following coculture treatment, GCs were collected and subjected to migration analysis using a Transwell system. A cell suspension containing 1.0 × 10^5^ cells in 100 μL serum‐free DMEM was loaded into the upper chamber, and the lower compartment was filled with 500 μL complete medium. After 6 h of incubation, nonmigratory cells were removed from the upper membrane surface. The migrated cells on the lower membrane surface were fixed with 4% paraformaldehyde, stained with 0.1% crystal violet solution, and quantified under a microscope at 400 × magnification.

### 2.14. Flow Cytometric Analysis

The collected cell populations were pelleted, rinsed, and subsequently resuspended to achieve a final concentration of 1 × 10^7^ cells per mL. Blank controls and isotype controls were established for the experiment. Primary antibodies, CD68 and CD86, were added to the cell samples for incubation to ensure sufficient antibody‐protein binding. This was followed by incubation with a secondary antibody conjugated to a fluorescent dye. After washing, the cells were resuspended, and M1 cell subpopulation (CD68^+^/CD86^+^) was measured using FACSCanto II (BD Biosciences). Negative isotypes were used for gating.

### 2.15. RNA Isolation and Quantitative Real‐Time PCR (qPCR)

Total RNA was isolated from cell samples using the TRIzol reagent (Invitrogen) following the manufacturer’s protocol. Sequence‐specific primers were manufactured by Sangon Biotech (Shanghai). Reverse transcription was performed using 500 ng total RNA as a template. Quantitative PCR amplification was carried out in triplicate using a miRNA‐specific SYBR Green master mix (Vazyme Biotech, Cat# MQ101) on a QuantStudio 5 Real‐Time PCR System (Applied Biosystems). Relative expression levels were calculated using the comparative threshold cycle (2^−ΔΔCt^) method.

### 2.16. Statistical Analysis

All statistical analyses and image creation were conducted using GraphPad Prism software. Two‐group comparisons employed unpaired two‐tailed *t*‐tests, while multigroup analyses utilized one‐way ANOVA. For post hoc testing following significant ANOVA results (*p* < 0.05), Tukey’s honestly significant difference (HSD) test was applied for all pairwise comparisons. Continuous variables were expressed as mean ± SD. The probability values below 0.05 were considered statistically significant for all tests.

## 3. Results

### 3.1. Hemin Promotes NLRP3 Inflammasome Activation in BMECs in a Dose‐Dependent Manner and Mediates GCs Recruitment and M1 Polarization

**Figure 1:**
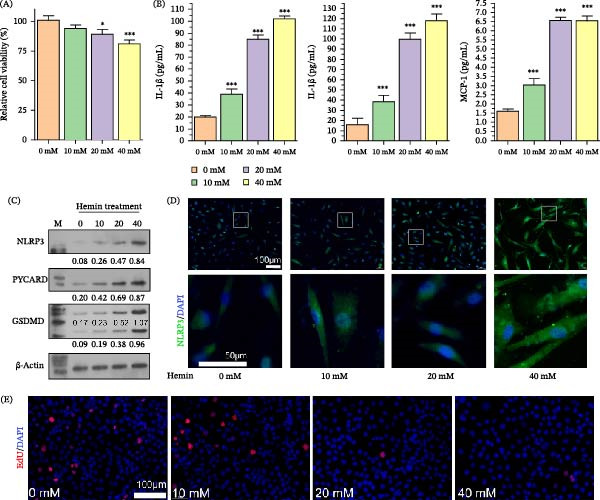
Hemin induces NLRP3 inflammasome activation and cytokine production in BMECs. (A) The cytotoxicity of hemin was evaluated by CCK8 assay. (B) ELISA results depicting increased levels of IL‐1β, IL‐18, and MCP‐1 in the cell culture supernatant corresponding to increasing hemin doses. (C) WB analysis demonstrating dose‐dependent upregulation of NLRP3, PYCARD, and an increase in GSDMD‐N protein levels with higher hemin concentrations. (D) Immunofluorescence images demonstrating enhanced punctate expression of NLRP3 in BMECs posthemin treatment. (E) Quantitative analysis of BMEC proliferation revealing a decline with increasing hemin concentrations.  ^∗^ *p* < 0.05,  ^∗∗∗^ *p* < 0.001 versus 0 mM group.

To explore the behavior of BMECs after ICH infection, experiments were conducted to treat BMECs with hemin. CCK‐8 assays revealed that hemin concentrations up to 40 mM only induced slight cytotoxicity in BMECs (Figure 1A). ELISA of the cell culture supernatant revealed that increasing hemin concentrations dose‐dependently increased proinflammatory factor levels (Figure 1B). Elevated hemin concentrations induced a dose‐responsive upregulation of key NLRP3 inflammasome elements, including NLRP3 and ASC (PYCARD), as well as GSDMD‐N (Figure 1C). IF analysis demonstrated that hemin treatment upregulated the expression of NLRP3 in BMECs, displaying a punctate pattern (Figure 1D). Additionally, hemin treatment was associated with a declining trend in the proliferation activity of BMECs (Figure 1E).

**Figure 2:**
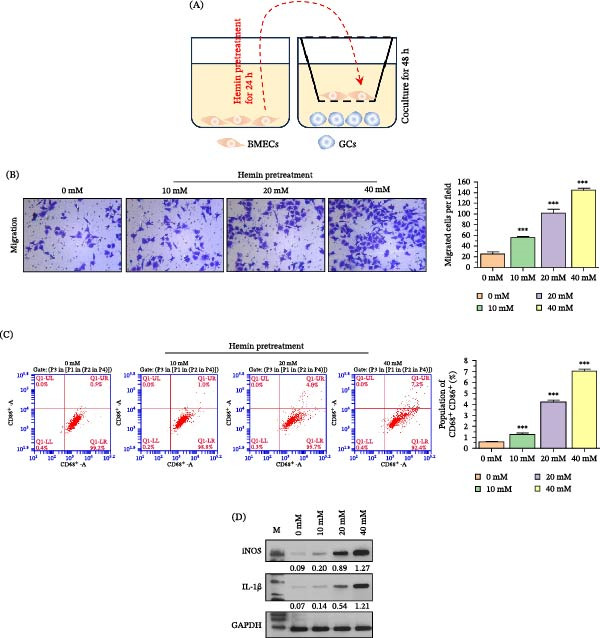
Hemin augments microglial migration and M1 polarization in cocultured BMECs. (A) Hemin‐pretreated BMECs were cocultured with GCs for 48 h. (B) Transwell assay data displaying enhanced microglial migration capability following hemin treatment in a dose‐dependent manner. (C) Flow cytometry results indicating increased M1 polarization of microglia (CD68^+^/CD86^+^) when cocultured with hemin‐treated BMECs. (D) Increasing hemin concentration further increases M1 polarization markers, including iNOS and IL‐1β protein levels.  ^∗∗∗^ *p* < 0.001 versus 0 mM group.

BMECs treated with hemin were cocultured with microglia to detect the migration and M1 polarization of GCs (Figure 2A). Hemin treatment significantly enhanced the migration ability of GCs with a dose‐dependent effect (Figure 2B). The GCs polarization detected by flow cytometry revealed that hemin treatment of BMECs promoted M1 polarization of GCs (Figure 2C). WB analysis also revealed elevated protein expression of iNOS and IL‐1βby by hemin stimulation (Figure 2D).

### 3.2. NLRP3 Inflammasome and Exosomes Mediate the Enhanced Migration and M1 Polarization of GCs by BMECs Pretreated With Hemin

**Figure 3:**
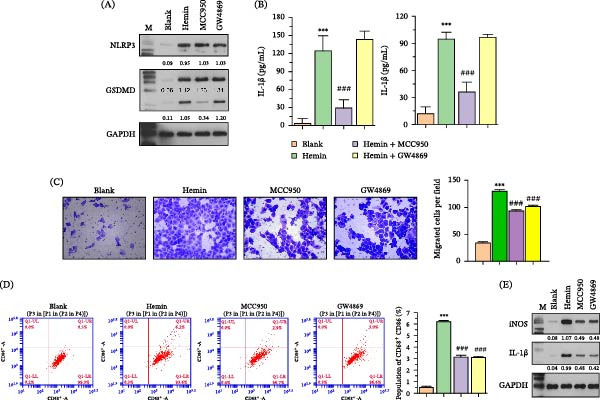
MCC950 inhibits NLRP3 inflammasome activation and affects cell migration and polarization. (A) WB analyses depicting decreased levels of GSDMD‐N in MCC950‐treated BMECs. (B) ELISA results displaying reduced levels of IL‐1β and IL‐18 following MCC950 treatment. (C) Migration assays revealing a significant reduction in the migration ability of BMECs and GCs with MCC950 and a further decrease when combined with GW4869. (D) Flow cytometry measurements indicating a decrease in M1 microglial polarization in cocultures treated with MCC950 and the reversal of hemin‐induced M1 polarization by GW4869. (E) WB confirmation of the reduced expression of iNOS and IL‐1β with GW4869 treatment in hemin‐stimulated cocultured GCs.  ^∗∗∗^ *p* < 0.001 versus blank group; ###*p* < 0.001 versus hemin group.

To explore the functional involvement of the NLRP3 inflammasome and exosomes in BMEC‐mediated GCs migration and polarization, BMECs were pretreated with hemin, NLRP3‐specific inhibitor MCC950, and the exosome release inhibitor, GW4869. Figure 3A,B demonstrate that MCC950 significantly suppressed NLRP3 inflammasome activation, reduced GSDMD‐N levels, and downregulated proinflammatory factor (IL‐1β and IL‐18) levels. Intriguingly, pretreatment of BMECs with MCC950 and GW4869 significantly reduced the migration ability of cocultured microglia (Figure 3C). Flow cytometry analysis demonstrated that hemin pretreatment of BMECs resulted in a significant increase in M1 polarization of GCs, which was reversed by pretreatment of BMECs with MCC950 and GW4869 (Figure 3D). WB experiments confirmed that the change in M1 polarization biomarkers (iNOS and IL‐1β) was consistent with flow cytometry results (Figure 3E).

### 3.3. Effect of NLRP3 Inflammasome on BMEC‐GCs Microenvironment Through Exosomes

**Figure 4:**
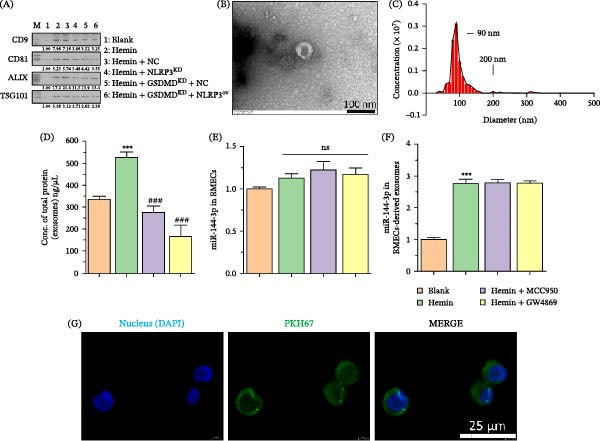
NLRP3 inflammasome impacts exosome characteristics and miR‐144‐3p content in BMEC‐derived exosomes. (A) WB validation of exosomal markers CD9, CD81, Alix, and TSG101 in BMEC‐derived exosomes. (B, C) TEM images displaying the double‐layered membrane structure of exosomes with an approximate diameter of 100 nm. (D) Quantitative analysis of total exosomal protein concentration in BMECs treated with hemin, MCC950, and GW4869. (E) miR‐144‐3p expression levels in BMECs following treatment with hemin, MCC950, or GW4869. (F) miR‐144‐3p levels in exosomes isolated from BMECs treated with hemin, MCC950, or GW4869. (G) Fluorescence microscopy images depicting the internalization of PKH67‐labeled BMEC‐derived exosomes by GCs.  ^∗∗∗^ *p* < 0.001 versus blank group; ###*p* < 0.001 versus hemin group.

To elucidate the role of exosomes in the BMEC‐GC environment and investigate whether NLRP3 activation influences them, BMEC‐derived exosomes were isolated, and relevant experiments were conducted. Initially, the isolated exosomes were identified using WB, which indicated that CD9, CD81, Alix, and TSG101 were present in these exosomes (Figure 4A). TEM demonstrated the presence of characteristic bilayer membrane vesicles exhibiting a typical exosomal morphology, with particle sizes averaging around 100 nm in diameter (Figure 4B,C). We investigated whether NLRP3 inflammasome activation affects exosomes. To evaluate exosome secretion, total exosomal protein was detected in different groups of BMECs. Hemin‐treated BMECs significantly upregulated exosomal protein concentrations, whereas MCC950 and GW4869 inhibitors significantly decreased BMEC‐derived exosomal protein concentrations (Figure 4D). Consequently, we inferred that the NLRP3 inflammasome promotes the release of BMEC‐derived exosomes. Moreover, we demonstrated that neither hemin nor MCC950 nor GW4869 altered miR‐144‐3p expression levels in BMECs; instead, hemin significantly elevated miR‐144‐3p levels in exosomes (isolated at equivalent exosomal protein concentrations) (Figure 4E,F). Finally, PKH67‐labeled BMEC‐derived exosomes were cocultured with GCs, and fluorescence signals observed within the GCs confirmed exosome uptake (Figure 4G).

### 3.4. GSDMD‐Mediated Exosomes Release From BMECs Play a Role in the BMEC‐GCs Environment

**Figure 5:**
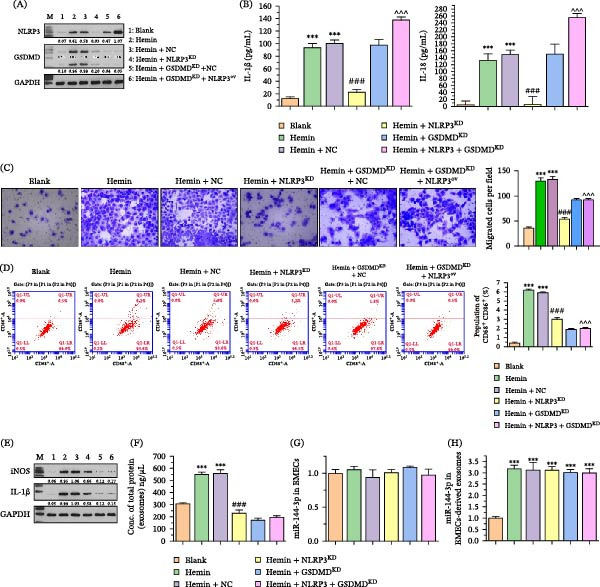
Effects of NLRP3 and GSDMD manipulation on IL‐1β, IL‐18, GC migration, polarization, and exosomal protein concentrations. (A) WB analysis displaying GSDMD‐N expression upon NLRP3 knockdown or overexpression in BMECs. (B) ELISA assays depicting IL‐1β and IL‐18 levels in BMECs with altered NLRP3 or GSDMD expression. (C) Migration assays demonstrate changes in GC migration capability following NLRP3 or GSDMD alteration in BMECs. (D) Flow cytometry analysis of GC polarization following NLRP3 knockdown, overexpression, and GSDMD interference. (E) Quantitative data on M1 polarization in GCs under various BMEC conditions. (F) Measurement of exosomal protein concentration in BMECs after modulation of NLRP3 and GSDMD expression. (G) miR‐144‐3p expression levels in BMECs with NLRP3 and GSDMD manipulation. (H) miR‐144‐3p abundance in exosomes derived from BMECs with altered NLRP3 and GSDMD expression.  ^∗^ *p* < 0.05 versus control group.  ^∗∗∗^ *p* < 0.001 versus blank group; ###*p* < 0.001 versus hemin + NC group; ^^^ *p* < 0.001 versus hemin + GSDMD ^KD^ group.

A previous study demonstrated that the NLRP3 inflammasome promotes exosome release and influences the recruitment and polarization of GCs, though the underlying regulatory mechanisms remain poorly characterized. To address this knowledge gap, we manipulated NLRP3 and GSDMD expression in BMECs and conducted experimental tests on BMECs and GCs. Our findings demonstrated that NLRP3 knockdown markedly suppressed GSDMD‐N terminal fragment production in BMECs and reduced the levels of proinflammatory factors in the cells. Conversely, NLRP3 overexpression elevated the levels of IL‐1β and IL‐18 in the cells (Figure 5A,B). Additionally, interference with GSDMD alone demonstrated limited effects on IL‐1β and IL‐18 levels in BMECs. NLRP3 knockdown and GSDMD interference in BMECs reduced the migration ability and M1 polarization of cocultured GCs. Notably, NLRP3 overexpression failed to reverse the reduction in GCs migration ability and M1 polarization caused by interference with GSDMD (Figure 5C–E). These findings indicate that the NLRP3 inflammasome‐dependent maturation of GSDMD plays a pivotal role for BMECs‐mediated GCs polarization and recruitment. Furthermore, we isolated exosomes from different groups of BMECs and found that knockdown of NLRP3 and GSDMD significantly reduced the protein concentration of exosomes in BMECs, indicating a decrease in exosome release (Figure 5F). Moreover, we found that NRLP3 overexpression alone could not reverse the effect of GSDMD knockdown, revealing the necessity of GSDMD. Interestingly, we found that neither the expression nor the abundance of miR‐144‐3p in BMEC‐derived exosomes showed selective modulation following the genetic alteration of NLRP3 and GSDMD in BMECs (Figure 5G,H). Therefore, the NLRP3/GSDMD pathway did not directly regulate miR‐144‐3p expression or exosomal abundance but may have mediated its transfer by controlling the total exosome release.

### 3.5. miR‐144‐3p Regulates BMECs‐Mediated GCs Polarization and Recruitment

**Figure 6:**
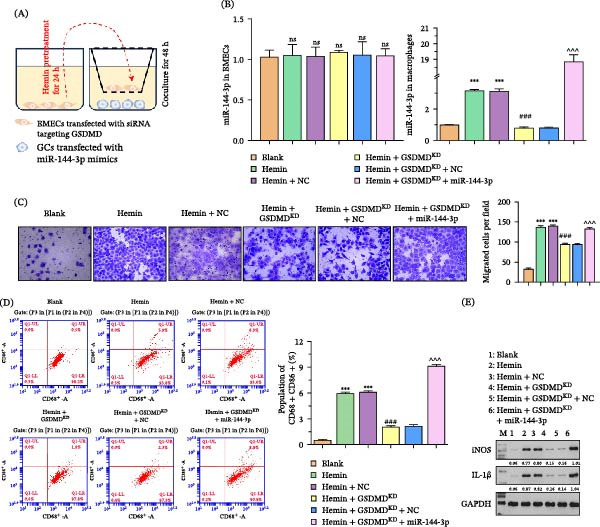
Impact of miR‐144‐3p on GC migration and polarization. (A) GSDMD siRNA was transfected into hemin pretreated BMECs, and miR‐144‐3p mimics were transfected into GCs. (B) Quantitative PCR analysis displaying levels of miR‐144‐3p in BMECs and GCs following hemin treatment and GSDMD siRNA transfection, with upregulation after miR‐144‐3p mimic transfection. (C) Transwell migration assays indicating restoration of GC migration after miR‐144‐3p overexpression in the presence of GSDMD siRNA. (D) Flow cytometry data illustrating that miR‐144‐3p overexpression counteracts the GSDMD siRNA‐induced decrease in M1 polarization of GCs. (E) WB results revealing increased iNOS and IL‐1β expression levels following miR‐144‐3p overexpression in GCs.  ^∗∗∗^ *p* < 0.001 versus blank group; ###*p* < 0.001 versus hemin + NC group; ^^^ *p* < 0.001 versus hemin + GSDMD ^KD^ + NC group.

To confirm the functional role of miR‐144‐3p in GCs polarization, we performed siRNA‐mediated GSDMD knockdown in BMECs and overexpressed miR‐144‐3p in GCs (Figure 6A). Notably, while miR‐144‐3p levels in hemin‐pretreated BMECs were unchanged, a marked increase was observed in cocultured GCs after hemin treatment. Further analysis demonstrated that GSDMD silencing in BMECs led to reduced miR‐144‐3p expression in GCs, whereas transfection with miR‐144‐3p mimics restored and significantly elevated its levels (Figure 6B). Transwell and flow cytometry experiments revealed that miR‐144‐3p overexpression significantly reversed the GCs migration and M1 polarization inhibition caused by GSDMD knockdown in BMECs (Figure 6C,D). Simultaneously, the expression levels of iNOS and IL‐1β were enhanced due to miR‐144‐3p overexpression (Figure 6E).

### 3.6. Role of NLRP3/GSDMD/miR‐144‐3p Pathway is Confirmed in Rat ICH Model

**Figure 7:**
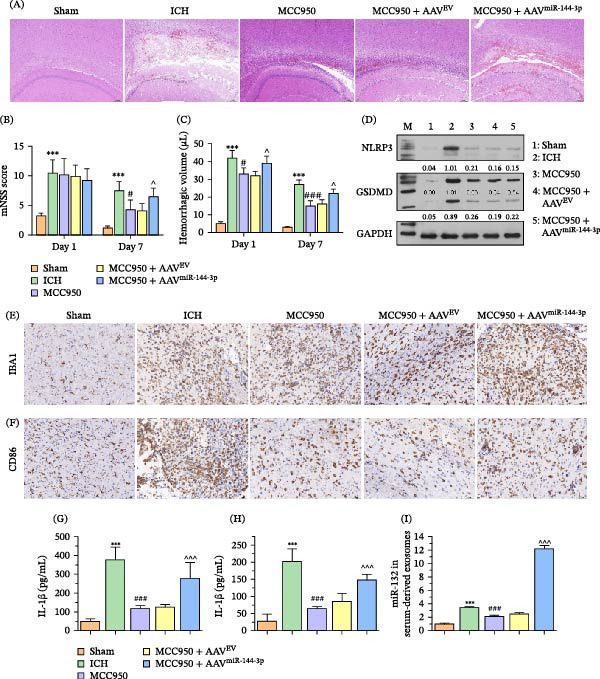
Effects of MCC950 and miR‐144‐3p overexpression on various markers in an ICH rat model. (A) HE staining was used to assess brain tissue injury. (B) Rat neural function was evaluated using the mNSS score. (C) Hemorrhagic volume detected using a spectrophotometric hemoglobin assay. (D) WB analysis of NLRP3 and GSDMD expression in rat brain tissue following MCC950 administration. (E, F) Immunohistochemical evaluation of IBA1 and CD86 distribution in the rat brains treated with MCC950. (G, H) ELISA quantifying IL‐18 and IL‐1β levels in the brain tissue of MCC950‐treated rats. (I) Quantification of miR‐144‐3p levels in serum EVs from ICH‐model rats.  ^∗∗∗^ *p* < 0.001 versus sham group; ^#^ *p* < 0.05 versus ICH group; ^###^ *p* < 0.001 versus ICH group; ^^^ *p* < 0.05 versus MCC950 + AAV^EV^ group; ^^^^^ *p* < 0.001 versus MCC950 + AAV^EV^ group.

The regulatory role of BMEC‐derived EVs in inducing the polarization and recruitment of GCs under the regulation of NLRP3 inflammasomes has been established in vitro. Therefore, we aimed to elucidate this process using an ICH rat model. As shown in Figure 7, MCC950 significantly mitigated the brain injury induced in the ICH model, whereas miR‐144‐3p overexpression aggravated the brain injury (Figure 7A). Next, the neurofunction was assessed by the mNSS score. Although neural damage was unaffected by MCC950 and miR‐144‐3p overexpression on day 1, it was significantly mitigated by MCC950 and aggravated by miR‐144‐3p overexpression on day 7 (Figure 7B). Additionally, the hemorrhagic volume was decreased by MCC950 and increased by miR‐144‐3p on days 1 and 7 (Figure 7C). Specifically, NLRP3 and GSDMD expression in the cerebral tissue were significantly downregulated by MCC950 (Figure 7D). Additionally, MCC950 suppressed the recruitment and M1 polarization of GCs, as evidenced by increased GCs biomarker CD86 and M1 GCs biomarker IBA1 expression. Intriguingly, these inhibitory effects were partially rescued upon miR‐144‐3p overexpression (Figure 7E,F). Furthermore, the ELISA assay demonstrated decreased proinflammatory factor levels in the MCC950 treatment group, indicating mitigated inflammatory injury by the NLRP3 inflammasomes in ICH. Notably, the effect of MCC950 was significantly reversed after injecting miR‐144‐3p overexpressing AAV, and elevated miR‐144‐3p levels were detected in animal serum EVs (Figure 7G–I).

## 4. Discussion

ICH, representing a particularly severe form of cerebrovascular accident, is associated with disproportionately high mortality and disability rates but no specific or effective treatment. Accordingly, understanding the molecular mechanisms underlying ICH is of great importance. As expected, exosomal miR‐144‐3p may regulate microglial M1 polarization, hence inducing inflammation in ICH. The following results can be drawn from this study: (1) NLRP3 inflammasome was activated in ICH, along with upregulation of GSDMD; (2) coculture with ICH endothelial cells promoted migration progression and M1 polarization of microglia cells; (32) exosomal miR‐144‐3p was markedly upregulated in ICH; (4) exosomes derived from ICH endothelial cells recruited microglia, enhancing their migration and M1 polarization; and (5) inhibition of exosomal miR‐144‐3p suppressed microglial migration and decreased M1 polarization both in vitro and in vivo. Herein, we aimed to delve into the mechanistic role of exosomal miRNA‐144‐3p in ICH through inflammation by recruiting microglial cells.

BMECs serve a crucial role in the BBB, functioning as a selective barrier that regulates the passage of substances from the bloodstream into the brain parenchyma. These cells are connected by tight junctions that maintain the integrity of the BBB, preventing the infiltration of potentially harmful substances while allowing essential nutrients to reach the brain [29]. In ICH, BMECs undergo pathological changes that can lead to BBB disruption, with a severe inflammatory response [30]. The NLRP3 inflammasome has been unequivocally established as a critical mediator of neuroinflammatory processes subsequent to ICH [31]. BMECs and microglia are significant participants in the inflammatory response. In this study, NLRP3 inflammasome activation was observed across both animal and cellular models. Enhanced expressions of NLRP3, PYCARD, and N‐GSDMD, along with a significant increase in proinflammatory factor levels, were observed in hemin‐treated BMECs.

GSDMD is a pivotal executor of pyroptotic cell death, serving as a key molecular link between cellular demise and inflammatory responses [32]. Upon cleavage by inflammatory caspases, including caspase‐1, it is split into two domains: an N‐terminal domain (N‐GSDMD) and a C‐terminal domain. N‐GSDMD is responsible for its biological activity. Upon cleavage, N‐GSDMD undergoes rapid translocation to the plasma membrane, where it forms transmembrane pores that disrupt membrane integrity and facilitate lytic cell death. This pore‐forming activity allows for the secretion of proinflammatory cellular contents, leading to inflammation and the characteristic cell swelling and lysis of pyroptosis [17]. The ability of N‐GSDMD to disrupt cellular membranes is critical for its role in the host’s defense mechanisms against infections and in the pathogenesis of several inflammatory diseases [33]. Our experimental findings demonstrate that NLRP3 knockdown or inhibitor treatment did not decrease the miR‐144‐3p levels. However, changed GSDMD expression significantly modulated the miR‐144‐3p level in exosomes. Consequently, we propose that N‐GSDMD is capable of regulating exosome secretion.

Exosomes serve as a promising miRNA delivery system due to their natural availability, biocompatibility, stability, and low immunogenicity of extracellular vesicles [34–36]. miRNAs are highly expressed in mammalian brain tissues compared to other organs [37] and have been reported to regulate brain diseases and pathological conditions [38, 39]. Inflammatory processes are found to be tightly linked to the regulatory role of miR‐144‐3p. During *Mycobacterium abscessus* infection, miR‐144‐3p upregulation is associated with increased pro‐inflammatory cytokines and exacerbated inflammation. This regulation suggests that miR‐144‐3p contributes to the pathological inflammation observed in patients and mouse models of the disease [20]. In osteoarthritis, both in vitro and animal model experiments have illustrated that miR‐144‐3p inversely regulates IL‐1β expression, subsequently downregulating inflammatory signaling pathways and ameliorating disease progression, highlighting the therapeutic potential of miR‐144‐3p modulation [40]. Currently, the function of miR‐144‐3p in ICH has never been directly confirmed. Our experimental data verified that miR‐144‐3p was enriched in exosomes from ICH BMECs.

Microglia, referred to as brain macrophages, are involved in neural environment maintenance, brain development, and response to injury and repair by recruiting M2 microglia/macrophages [41, 42]. Significant efforts have been made to regulate the M1/M2 polarization. Researchers have found that LPS preconditioning enhances functional recovery after spinal cord injury through selective promotion of M2 macrophage polarization [43]. Similarly, IL‐4 treatment has been shown to effectively induce M2 phenotype conversion and reduce the infarct size [44]. The present study found that M1 polarization was promoted when cocultured with ICH endothelial cells. Moreover, microglial cell migration was elevated. Shtaya et al. [45] reported that the size and morphology of microglia were gradually changed post‐ICH, which may be related to the phagocytic function. Ye et al. [46] revealed that thrombin‐mediated activation of the NLRP3 inflammasome during ICH progression involves ROS‐dependent TXNIP signaling, which subsequently triggers microglial apoptosis. These studies have also concluded that microglial cell progression is vital for advancing ICH. In this study, the in vitro ICH model promoted M1 polarization of microglia. Further experiments revealed that exosomal miR‐144‐3p, mediated by NLRP3‐GSDMD, was taken up by microglia, resulting in the regulation of microglial polarization. Although exosome uptake by microglia was demonstrated in vitro, technical limitations prevented its confirmation in the rat model, particularly for exosome‐delivered miR‐144‐3p. This represents a limitation of our study.

Due to the complexity of the intrabrain environment, ICH treatment has always been a clinical problem because of the difficulty in regenerating and sustaining neuritis. Compared with other treatments, exosomes may be more effective for the following reasons. BMSCs‐exosomes are small enough to pass through BBB to reach the injured tissues; exosomes serve as an ideal transmission carrier, protecting enzymes or RNAs from degradation and promoting their uptake by microglia [47].

## 5. Conclusion

In summary, this study revealed that the NLRP3/GSDMD pathway promotes the secretion of BMEC‐derived exosomes, which transfer miR‐144‐3p from BMECs to GCs, further inducing the migration and M1 polarization of GCs (Figure S1). The findings of this study underscore the critical involvement of the NLRP3 inflammasome in driving neuroinflammation following ICH and suggest potential therapeutic strategies targeting GC activity and inflammation through modulation by MCC950 and miR‐144‐3p.

## Author Contributions

Conception and design: Hao Yin. Administrative support: Hao Yin and Zhongying Ran. Provision of study materials or patients: Tao Luo. Collection and assembly of data: Zexin Jin. Data analysis and interpretation: Ying Tan. Manuscript writing: All authors. Funding acquisition: Jun Ma.

## Funding

This research is supported by the Health Commission of Guizhou Province Scientific Research Projects (Grant gzwkj2023‐156 to Hao Yin, Grant gzwkj2023‐159 to Jun Ma) and Guizhou Provincial Basic Research Program (Natural Science) (Grant ZK [2024] 473 to Jun Ma, Grant ZK [2025]491 to Hao Yin).

## Disclosure

All authors contributed to the final approval of manuscript.

## Ethics Statement

The study was approved by the ethics committee of the Guizhou Provincial People’s Hospital (Number SYXK2018‐0003).

## Conflicts of Interest

The authors declare no conflicts of interest.

## Supporting Information

Additional supporting information can be found online in the Supporting Information section.

## Supporting information


**Supporting Information** Figure S1: Graphical abstract. NLRP3/GSDMD pathway promotes the secretion of BMEC‐derived exosomes, which transfer miR‐144‐3p from BMECs to GCs, further inducing the migration and M1 polarization of GCs.

## Data Availability

The data that support the findings of this study are available from the corresponding author upon reasonable request.

## References

[1] Keep R. F. , Hua Y. , and Xi G. , Intracerebral Haemorrhage: Mechanisms of Injury and Therapeutic Targets, The Lancet Neurology. (2012) 11, no. 8, 720–731, 10.1016/S1474-4422(12)70104-7, 2-s2.0-84863863921. 22698888 PMC3884550

[2] Van Asch C. J. , Luitse M. J. , and Rinkel G. J. , et al.Incidence, Case Fatality, and Functional Outcome of Intracerebral Haemorrhage Over Time, According to Age, Sex, and Ethnic Origin: A Systematic Review and Meta-Analysis, The Lancet Neurology. (2010) 9, no. 2, 167–176, 10.1016/S1474-4422(09)70340-0, 2-s2.0-76249112303. 20056489

[3] Xiong X.-Y. , Liu L. , and Yang Q.-W. , Functions and Mechanisms of Microglia/Macrophages in Neuroinflammation and Neurogenesis After Stroke, Progress in Neurobiology. (2016) 142, 23–44, 10.1016/j.pneurobio.2016.05.001, 2-s2.0-84974626058. 27166859

[4] Bai Q. , Xue M. , and Yong V. W. , Microglia and Macrophage Phenotypes in Intracerebral Haemorrhage Injury: Therapeutic Opportunities, Brain. (2020) 143, no. 5, 1297–1314, 10.1093/brain/awz393. 31919518

[5] Xu J. , Duan Z. , and Qi X. , et al.Injectable Gelatin Hydrogel Suppresses Inflammation and Enhances Functional Recovery in a Mouse Model of Intracerebral Hemorrhage, Frontiers in Bioengineering Biotechnology. (2020) 8, 82020–82785, 10.3389/fbioe.2020.00785. PMC7371925 32760708

[6] Shao G. , Zhou C. , and Ma K. , et al.MiRNA-494 Enhances M1 Macrophage Polarization via Nrdp1 in ICH Mice Model, Journal of Inflammation. (2020) 17, no. 1, 1–13, 10.1186/s12950-020-00247-3. 32351331 PMC7183644

[7] Cheng J. , Tang J.-C. , and Pan M. X. , l-Lysine Confers Neuroprotection by Suppressing Inflammatory Response via microRNA-575/PTEN Signaling After Mouse Intracerebral Hemorrhage Injury, Experimental Neurology. (2020) 327, 113214. 31987833 10.1016/j.expneurol.2020.113214

[8] Lian X. W. and Luo B. , Knockdown of NEAT1 Induced Microglial M2 Polarization via miR-374a-5p/NFAT5 Axis to Inhibit Inflammatory Response Caused by OGD/R, Acta Neurobiologiae Experimentalis. (2021) 81, no. 4, 362–374, 10.55782/ane-2021-035. 35014985

[9] Gharavi A. T. , Hanjani N. A. , and Movahed E. , et al.The Role of Macrophage Subtypes and Exosomes in Immunomodulation, Cellular Molecular Biology Letters. (2022) 27, no. 1, 10.1186/s11658-022-00384-y, 83. 36192691 PMC9528143

[10] Wang L. , Yi X. , and Xiao X. , et al.Exosomal miR-628-5p From M1 Polarized Macrophages Hinders m6A Modification of circFUT8 to Suppress Hepatocellular Carcinoma Progression, Cellular Molecular Biology Letters. (2022) 27, no. 1, 10.1186/s11658-022-00406-9. PMC9724320 36474147

[11] Forró T. , Bajkó Z. , and Bălașa A. , et al.Dysfunction of the Neurovascular Unit in Ischemic Stroke: Highlights on microRNAs and Exosomes as Potential Biomarkers and Therapy, International Journal of Molecular Sciences. (2021) 22, no. 11, 10.3390/ijms22115621, 5621. 34070696 PMC8198979

[12] Zhang M. , Wu Q. , and Tang M. , et al.Exosomal Mir-3613-3p Derived From Oxygen–Glucose Deprivation-Treated Brain Microvascular Endothelial Cell Promotes Microglial M1 Polarization, Cellular Molecular Biology Letters. (2023) 28, no. 1, 10.1186/s11658-023-00432-1, 18. 36870962 PMC9985860

[13] Wan T. , Huang Y. , and Gao X. , et al.Microglia Polarization: A Novel Target of Exosome for Stroke Treatment, Frontiers in Cell Developmental Biology. (2022) 10, 10.3389/fcell.2022.842320. PMC8959940 35356292

[14] Zhang Z. , Zou X. , and Zhang R. , et al.Human Umbilical Cord Mesenchymal Stem Cell-Derived Exosomal miR-146a-5p Reduces Microglial-Mediated Neuroinflammation via Suppression of the IRAK1/TRAF6 Signaling Pathway After Ischemic Stroke, Sedentary Life and Nutrition. (2021) 13, no. 2, 3060–3079, 10.18632/aging.202466. PMC7880318 33479185

[15] Cypryk W. , Nyman T. A. , and Matikainen S. , From Inflammasome to Exosome-Does Extracellular Vesicle Secretion Constitute an Inflammasome-Dependent Immune Response?, Frontiers in Immunology. (2018) 9, 10.3389/fimmu.2018.02188, 2-s2.0-85054887942, 2188. 30319640 PMC6167409

[16] Wright S. S. , Kumari P. , and Fraile-Agreda V. , et al.Transplantation of Gasdermin Pores by Extracellular Vesicles Propagates Pyroptosis to Bystander Cells, Cell. (2025) 188, no. 2, 280–291, 10.1016/j.cell.2024.11.018. 39742811 PMC12272064

[17] Bulek K. , Zhao J. , and Liao Y. , et al.Epithelial-Derived Gasdermin D Mediates Nonlytic IL-1β Release During Experimental Colitis, The Journal of Clinical Investigation. (2020) 130, no. 8, 4218–4234, 10.1172/JCI138103. 32597834 PMC7410065

[18] Mouasni S. , Gonzalez V. , and Schmitt A. , et al.The Classical NLRP3 Inflammasome Controls FADD Unconventional Secretion Through Microvesicle Shedding, Cell Death & Disease. (2019) 10, no. 3, 10.1038/s41419-019-1412-9, 2-s2.0-85062058472. PMC6389912 30804327

[19] Bartel D. P. , Metazoan MicroRNAs, Cell. (2018) 173, no. 1, 20–51, 10.1016/j.cell.2018.03.006, 2-s2.0-85044007903. 29570994 PMC6091663

[20] Kim H. J. , Kim I. S. , and Lee S. G. , et al.MiR-144-3p is Associated With Pathological Inflammation in Patients Infected With Mycobacteroides Abscessus, Experimental & Molecular Medicine. (2021) 53, no. 1, 136–149, 10.1038/s12276-020-00552-0. 33473145 PMC8080579

[21] Sun G. , Lu Y. , and Zhao L. , et al.Hemin Impairs Resolution of Inflammation via microRNA-144-3p-Dependent Downregulation of ALX/FPR2, Transfusion. (2019) 59, no. 1, 196–206, 10.1111/trf.14991, 2-s2.0-85057813647. 30499593

[22] Qu P. , Xie X. , and Chi J. , et al.Circulating Exosomal miR-144-3p From Crohn’s Disease Patients Inhibits Human Umbilical Vein Endothelial Cell Function by Targeting FN1, Disease Markers. (2022) 2022, 10.1155/2022/8219557, 8219557. 35692876 PMC9184168

[23] Kung T. F. , Wilkinson C. M. , and Dirks C. A. , et al.Glibenclamide Does not Improve Outcome Following Severe Collagenase-Induced Intracerebral Hemorrhage in Rats, PLoS ONE. (2021) 16, no. 6, 10.1371/journal.pone.0252584, e0252584. 34081746 PMC8174736

[24] Chen R. , Xie Q. , and Xie L. , et al.Thioredoxin1 Binding Metastasis-Associated Lung Adenocarcinoma Transcript 1 Attenuates Inflammation and Apoptosis After Intracerebral Hemorrhage, Aging and Disease. (2024) 15, no. 3, 1384–1397, 10.14336/AD.2023.0507. 37196136 PMC11081159

[25] Shi Z. M. , Jing J. J. , and Xue Z. J. , et al.Stellate Ganglion Block Ameliorated Central Post-Stroke Pain With Comorbid Anxiety and Depression Through Inhibiting HIF-1alpha/NLRP3 Signaling Following Thalamic Hemorrhagic Stroke, Journal of Neuroinflammation. (2023) 20, no. 1, 10.1186/s12974-023-02765-2, 82. 36944982 PMC10031944

[26] Chen J. , Li Y. , and Wang L. , et al.Therapeutic Benefit of Intravenous Administration of Bone Marrow Stromal Cells After Cerebral Ischemia in Rats, Stroke. (2001) 32, no. 4, 1005–1011, 10.1161/01.STR.32.4.1005, 2-s2.0-0035075998. 11283404

[27] Liew H. K. , Cheng H. Y. , and Huang L. C. , et al.Acute Alcohol Intoxication Aggravates Brain Injury Caused by Intracerebral Hemorrhage in Rats, Journal of Stroke and Cerebrovascular Diseases. (2016) 25, no. 1, 15–25, 10.1016/j.jstrokecerebrovasdis.2015.08.027, 2-s2.0-84960373666. 26387045

[28] Hamanaka G. , Kubo T. , and Ohtomo R. , et al.Microglial Responses after Phagocytosis: *Escherichia coli* Bioparticles, but not Cell Debris or Amyloid Beta, Induce Matrix Metalloproteinase-9 Secretion in Cultured Rat Primary Microglial Cells, Glia. (2020) 68, no. 7, 1435–1444, 10.1002/glia.23791. 32057146 PMC7256913

[29] Takata F. , Nakagawa S. , and Matsumoto J. , et al.Blood-Brain Barrier Dysfunction Amplifies the Development of Neuroinflammation Understanding of Cellular Events in Brain Microvascular Endothelial Cells for Prevention and Treatment of BBB Dysfunction, Frontiers in Cellular Neuroscience. (2021) 15, 661838. 34588955 10.3389/fncel.2021.661838 PMC8475767

[30] Lizano P. , Pong S. , and Santarriaga S. , et al.Brain Microvascular Endothelial Cells and Blood-Brain Barrier Dysfunction in Psychotic Disorders, Molecular Psychiatry. (2023) 28, no. 9, 3698–3708, 10.1038/s41380-023-02255-0. 37730841

[31] Wang T. , Nowrangi D. , and Yu L. , et al.Activation of Dopamine D1 Receptor Decreased NLRP3-Mediated Inflammation in Intracerebral Hemorrhage Mice, Journal of Neuroinflammation. (2018) 15, no. 1, 10.1186/s12974-017-1039-7, 2-s2.0-85042511609, 2. 29301581 PMC5753458

[32] Karmakar M. , Minns M. , and Greenberg E. N. , et al.N-GSDMD Trafficking to Neutrophil Organelles Facilitates IL-1β Release Independently of Plasma Membrane Pores and Pyroptosis, Nature Communications. (2020) 11, no. 1, 10.1038/s41467-020-16043-9, 2212. PMC7200749 32371889

[33] Ding P. , Yang R. , and Li C. , et al.Fibroblast Growth Factor 21 Attenuates Ventilator-Induced Lung Injury by Inhibiting the NLRP3/Caspase-1/GSDMD Pyroptotic Pathway, Critical Care. (2023) 27, no. 1, 10.1186/s13054-023-04488-5. PMC10204208 37218012

[34] Samanta S. , Rajasingh S. , and Drosos N. , et al.Exosomes: New Molecular Targets of Diseases, Acta Pharmacologica Sinica. (2018) 39, no. 4, 501–513, 10.1038/aps.2017.162, 2-s2.0-85044785911. 29219950 PMC5888687

[35] Simeoli R. , Montague K. , and Jones H. R. , et al.Exosomal Cargo Including microRNA Regulates Sensory Neuron to Macrophage Communication After Nerve Trauma, Nature Communications. (2017) 8, no. 1, 10.1038/s41467-017-01841-5, 2-s2.0-85034950039, 1778. PMC5701122 29176651

[36] Lv Q. , Deng J. , and Chen Y. , et al.Engineered Human Adipose Stem-Cell-Derived Exosomes Loaded With miR-21-5p to Promote Diabetic Cutaneous Wound Healing, Molecular Pharmaceutics. (2020) 17, no. 5, 1723–1733, 10.1021/acs.molpharmaceut.0c00177. 32233440

[37] Sempere L. F. , Freemantle S. , and Pitha-Rowe I. , et al.Expression Profiling of Mammalian microRNAs Uncovers a Subset of Brain-Expressed microRNAs With Possible Roles in Murine and Human Neuronal Differentiation, Genome Biology. (2004) 5, no. 3, 1–11, 10.1186/gb-2004-5-3-r13. PMC395763 15003116

[38] Bhalala O. G. , Srikanth M. , and Kessler J. A. , The Emerging Roles of microRNAs in CNS Injuries, Nature Reviews Neurology. (2013) 9, no. 6, 328–339, 10.1038/nrneurol.2013.67, 2-s2.0-84878945439. 23588363 PMC3755895

[39] Moon J.-m. , Xu L. , and Giffard R. G. , Inhibition of microRNA-181 Reduces Forebrain Ischemia-Induced Neuronal Loss, Journal of Cerebral Blood Flow Metabolism. (2013) 33, no. 12, 1976–1982, 10.1038/jcbfm.2013.157, 2-s2.0-84889097694. 24002437 PMC3851907

[40] Lin Y. , Ko C. , and Liu S. , et al.miR-144-3p Ameliorates the Progression of Osteoarthritis by Targeting IL-1β: Potential Therapeutic Implications, Journal of Cellular Physiology. (2021) 236, no. 10, 6988–7000, 10.1002/jcp.30361. 33772768

[41] Ransohoff R. M. and Cardona A. E. , The Myeloid Cells of the Central Nervous System Parenchyma, Nature. (2010) 468, no. 7321, 253–262, 10.1038/nature09615, 2-s2.0-78149435065. 21068834

[42] Zhang Z. , Zhang Z. , and Lu H. , et al.Microglial Polarization and Inflammatory Mediators After Intracerebral Hemorrhage, Molecular Neurobiology. (2017) 54, no. 3, 1874–1886, 10.1007/s12035-016-9785-6, 2-s2.0-84958739618. 26894396 PMC4991954

[43] Hayakawa K. , Okazaki R. , and Morioka K. , et al.Lipopolysaccharide Preconditioning Facilitates M2 Activation of Resident Microglia After Spinal Cord Injury, Journal of Neuroscience Research. (2014) 92, no. 12, 1647–1658, 10.1002/jnr.23448, 2-s2.0-84911987395. 25044014

[44] Zhao X. , Wang H. , and Sun G. , et al.Neuronal Interleukin-4 as a Modulator of Microglial Pathways and Ischemic Brain Damage, Journal of Neuroscience. (2015) 35, no. 32, 11281–11291, 10.1523/JNEUROSCI.1685-15.2015, 2-s2.0-84939122706. 26269636 PMC4532758

[45] Shtaya A. , Bridges L. R. , and Esiri M. M. , et al.Rapid Neuroinflammatory Changes in Human Acute Intracerebral Hemorrhage, Annals of Clinical Translational Neurology. (2019) 6, no. 8, 1465–1479, 10.1002/acn3.50842, 2-s2.0-85070648409. 31402627 PMC6689697

[46] Ye X. , Zuo D. , and Yu L. , et al.ROS/TXNIP Pathway Contributes to Thrombin Induced NLRP3 Inflammasome Activation and Cell Apoptosis in Microglia, Biochemical Biophysical Research Communications. (2017) 485, no. 2, 499–505, 10.1016/j.bbrc.2017.02.019, 2-s2.0-85013486076. 28202418

[47] Fan Y. , Chen Z. , and Zhang M. , Role of Exosomes in the Pathogenesis, Diagnosis, and Treatment of Central Nervous System Diseases, Journal of Translational Medicine. (2022) 20, no. 1, 10.1186/s12967-022-03493-6, 291. 35761337 PMC9235237

