# Supplementary figures and images for: Role of BMEC‐Derived Exosomal miR‐144‐3p in Microglial Polarization: Unveiling the NLRP3‐GSDMD Pathway in Intracerebral Hemorrhage
Source: Mediators Inflamm. 2026 May 26;2026:6142465. doi: 10.1155/mi/6142465 (PMC13202724; doi:10.1155/mi/6142465)

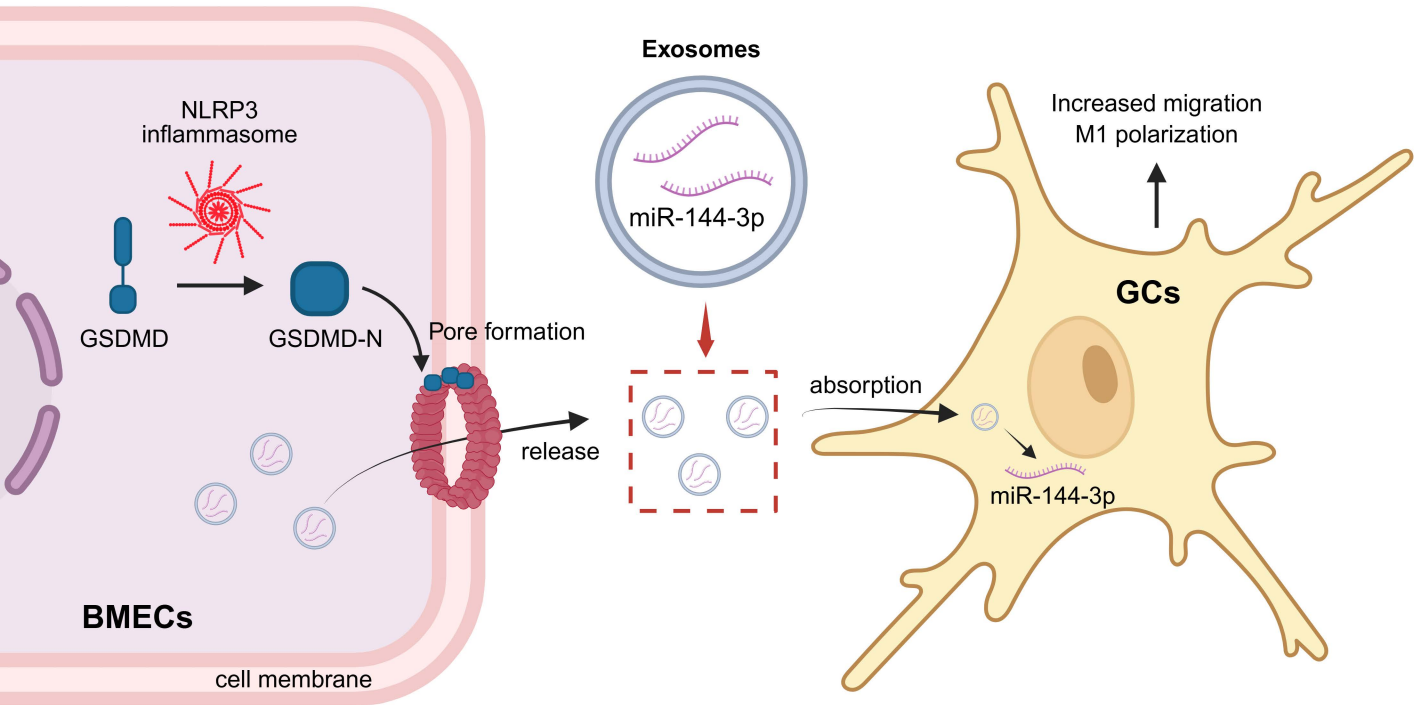

Supplement: Supplementary file 1 — Supporting Information Figure S1: Graphical abstract. NLRP3/GSDMD pathway promotes the secretion of BMEC‐derived exosomes, which transfer miR‐144‐3p from BMECs to GCs, further inducing the migration and M1 polarization of GCs. [file MI-2026-6142465-s001.pdf]
